# Transcriptional portrait of *M. bovis* BCG during biofilm production shows genes differentially expressed during intercellular aggregation and substrate attachment

**DOI:** 10.1038/s41598-020-69152-2

**Published:** 2020-07-28

**Authors:** Mario Alberto Flores-Valdez, Michel de Jesús Aceves-Sánchez, Eliza J. R. Peterson, Nitin Baliga, Jorge Bravo-Madrigal, Miguel Ángel De la Cruz-Villegas, Miguel A. Ares, Sarah Born, Martin Voskuil, Nayeli Areli Pérez-Padilla, Mirna Burciaga-Flores, Tanya Amanda Camacho-Villegas, María Guadalupe Espinoza-Jorge

**Affiliations:** 1 Biotecnología Médica y Farmacéutica, Centro de Investigación y, Asistencia en Tecnología y diseño del Estado de Jalisco, A.C., Av. Normalistas 800, Col. Colinas de La Normal, 44270 Guadalajara, Jalisco Mexico; 2 Institute for Systems Biology, Seattle, WA 98109 USA; 3 Unidad de Investigación Médica en Enfermedades Infecciosas y Parasitarias, Centro Médico Nacional (CMN) Siglo XXI, Instituto Mexicano de Seguro Social (IMSS), Mexico City, Mexico; 4 Department of Immunology and Microbiology, University of Colorado School of Medicine, Aurora, CO 80045 USA

**Keywords:** Microbiology, Bacteria, Bacterial transcription

## Abstract

*Mycobacterium tuberculosis* and *M. smegmatis* form drug-tolerant biofilms through dedicated genetic programs. In support of a stepwise process regulating biofilm production in mycobacteria, it was shown elsewhere that *lsr2* participates in intercellular aggregation, while *groEL1* was required for biofilm maturation in *M. smegmatis*. Here, by means of RNA-Seq, we monitored the early steps of biofilm production in *M. bovis* BCG, to distinguish intercellular aggregation from attachment to a surface. Genes encoding for the transcriptional regulators *dosR* and *BCG0114* (*Rv0081*) were significantly regulated and responded differently to intercellular aggregation and surface attachment. Moreover, a *M. tuberculosis* H37Rv deletion mutant in the *Rv3134c-dosS-dosR* regulon, formed less biofilm than wild type *M. tuberculosis*, a phenotype reverted upon reintroduction of this operon into the mutant. Combining RT-qPCR with microbiological assays (colony and surface pellicle morphologies, biofilm quantification, Ziehl–Neelsen staining, growth curve and replication of planktonic cells), we found that *BCG0642c* affected biofilm production and replication of planktonic BCG, whereas *ethR* affected only phenotypes linked to planktonic cells despite its downregulation at the intercellular aggregation step. Our results provide evidence for a stage-dependent expression of genes that contribute to biofilm production in slow-growing mycobacteria.

## Introduction

In nature, microbial species are often found within a matrix, forming multicellular communities that attach to surfaces or air–liquid interfaces, called biofilms^1^. Biofilms are relevant to human health, as a majority of bacterial pathogens employ these structures to modify the host response^2^ contributing to persistence^3^. In this regard, a link exists between in vitro biofilm production and in vivo persistence for BCG^4^ and *M. tuberculosis*^5^. Biofilm formation occurs via a series of well-defined steps. These include the attachment of single-cell planktonic microbes onto a substratum; aggregation and growth of the adherent cells into three-dimensionally organized structures; and encapsulation of the structures by a self-produced matrix of extracellular polymeric substance^1^.

*Mycobacterium tuberculosis* and *M. smegmatis* form drug-tolerant biofilms through dedicated genetic programs^6,7^. In support of a stepwise process regulating biofilm production in mycobacteria, it was recently shown that in *M. smegmatis*, *lsr2* participates in intercellular aggregation, while *groEL1* was required for biofilm maturation^1^. Additionally, it was found that multiple genes that are necessary for fitness of *M. tuberculosis* cells within biofilms, were also implicated in tolerance to a diverse set of stressors and antibiotics^8^, something not observed for planktonic cells, further supporting a role for *M. tuberculosis* biofilms in drug tolerance^7^.

To date, a number of genes have been shown to affect the capacity of mycobacteria to produce biofilms in vitro, with few reports describing their specific participation in the stepwise process of regulating biofilm production. Here, by means of whole transcriptome analysis, we monitored the early steps of biofilm production in *M. bovis* BCG, to distinguish intercellular aggregation from attachment to a surface. We identified a number of genes being differentially expressed at these stages, including genes encoding for the transcriptional regulators linked to oxygen availability, *dosR* and *BCG0114* (*Rv0081*), which were expressed in a temporal order during mycobacterial biofilm formation. Our results also provide a potential explanation for a stage-dependent expression of additional genes previously reported to contribute to biofilm production in mycobacteria and suggest new targets that can be assessed for their particular contribution to this phenotype.

## Results

### Transcriptional profiling during intercellular aggregation and substrate attachment

Our model of biofilm production by BCG consists of four distinct stages based on visual inspection as cultures progressed in Sauton medium from planktonic cells to mature biofilms. There, BCG starts as free-swimming bacteria (24 h) that in the absence of detergent, forms microcolonies of aggregated cells that can be readily visible (7 days). Later, these aggregates attach to the plastic wells (10 days), to finally produce mature surface pellicles that cover all the air–liquid interphase as well as part of the plastic wells (14 days). We previously demonstrated that it is possible to visually detect these steps during BCG biofilm formation at these time points^9^. To reduce potential variation from experiment to experiment, we always started biofilm cultures with cells adjusted at OD600nm 0.03.

To investigate how BCG responds to intercellular aggregation, differential gene expression analysis was used to compare the transcriptome of 7-day-old cultures (visible intercellular aggregation) as compared with 24 h cultures (basal transcriptome of BCG planktonic cells). Next, we interrogated how BCG specifically responds to substrate attachment, by comparing the transcriptome of 10 days-old cultures (visible attachment to the plastic walls) as compared with 7 days cultures (visible intercellular aggregation). The BCG Pasteur 1173P2 genome, used as a reference, has 4,109 protein and RNA-encoding genes. In our assays, we were able to detect differential gene expression [considered as significant (when both Log_2_-fold change ≥ 1 or ≤ − 1 plus *p* < 0.05) or not] when comparing intercellular aggregation versus growth as planktonic cells. We found mostly gene downregulation during this transition [1503 upregulated (37.5% of the coding potential), 2,605 downregulated (65%), Supplementary Table 1]. For substrate attachment, as compared with intercellular aggregation, there was less of a biased response [1975 (49.3%) upregulated and 2,132 (53.2%) downregulated, Supplementary Table 1].

For illustrative purposes, the 30 most significantly up- or down-regulated genes are shown in Table 2. Among the most upregulated genes with a known function, we found *BCG3184c* (*Rv3160c*, probable TetR-family transcriptional regulator), *sigE*, *fadE23* (fatty-acid-CoA ligase, involved in sulfolipid production)^10^, *hupB* (DNA binding protein), *BCG3929* (*Rv3866*, *espG*), *ppsC* (involved in PDIM synthesis), *BCG1191* (*Rv1130*, *prpD*, 2-methylcitrate dehydratase), and *BCG1826* (*Rv1794*, part of the ESX-5 secretion system)^11^. Of note, 9 out of the 30 most downregulated genes in the transition from planktonic cells to intercellular aggregation were members of the DosR regulon (Dormancy Survival Regulon). The DosR-regulon is composed of 48 genes that respond to in vitro microaerophilic or hypoxic conditions as well as exposure to nitric oxide or replication within macrophages^12,13^. Genes *BCG0115* (*Rv0082*), *BCG0112* (*Rv0079*), *BCG0114* (*Rv0081*), *BCG0113* (*Rv0080*), *BCG3157c* (*Rv3134c*), *TB31.7* (*Rv2623*), *hspX* (*Rv2031c*), *BCG2049c* (*Rv2030c*), and *BCG3154* (*Rv3131*) were downregulated when BCG changed from planktonic cells to the intercellular aggregation step.

Interestingly, 14 out of the 30 most upregulated genes in the transition from intercellular aggregation to surface attachment were also part of the DosR regulon: *BCG0112* (*Rv0079*), *BCG2051* (*acg*), *BCG2653c* (*Rv2626c*), *BCG3157c* (*Rv3134c*), *BCG1777* (*Rv1738*), *BCG1772c* (*Rv1733c*), *TB31.7* (*Rv2623*), *BCG0115* (*Rv0082*), *BCG0614* (*Rv0569*), *hspX* (*Rv2031c*), *BCG3154* (*Rv3131*), and *BCG2049c* (*Rv2030c*). Genes downregulated when BCG changed from intercellular aggregation to surface attachment were mostly encoding for hypothetical, conserved hypothetical, or membrane-associated proteins, with the exception of the one encoding for DNA-directed RNA polymerase subunit α, *rpoA*.

The total of differentially expressed genes showing a statistically significant difference (Log_2_-fold change ≥ 1 or ≤ − 1, *p* < 0.05) with respect to the reference, previous growth stage are shown in Supplementary Table 2 [upregulated during intercellular aggregation, 248 genes (6.2%)], Supplementary Table 3 [downregulated during intercellular aggregation, 764 genes (19.1%)], Supplementary Table 4 [upregulated during substrate attachment, 474 genes (11.8%)], and Supplementary Table 5 [downregulated during substrate attachment, 683 genes (17%)].

Pang et al. described some genes as relevant for biofilm production in *M. tuberculosis*, such as *PE1*, *nirB*, *PPE*5, *mycP1*, and *pks1*, among others^14^, although their temporal requirement during biofilm production was not elucidated. Later, *lsr2* was implicated in intercellular aggregation^1^. We found that *PE1*, *nirB*, and *lsr2* were moderately upregulated (FC = 0.6, 0.7, and 0.95 Log_2_, respectively, Supplementary Table 1) during the transition from planktonic to intercellular aggregation, while their expression moderately decreased (FC = − 0.88, − 0.64, and − 0.8 Log_2_, respectively, Supplementary Table 1) during substratum attachment. In both instances, the FC set up in our screening to find differentially expressed genes (Log_2_ ≥ 1) was not reached, and therefore these 3 genes were not considered as DE in our analyses, although we acknowledge that the p-value found for these genes was statistically significant and below the threshold of *p* ≤ 0.05. As for *PE5* and *pks1*, neither of these genes complied with FC and *p* value criteria set up here to be considered as DE.

GroEL1 was reported to be required for biofilm production in *M. smegmatis* via its binding to KasA and regulation of mycolic acids synthesis and biofilm maturation^6^. On the other hand, in BCG GL2, deletion of *groEL1* produced thinner surface pellicles, devoid of PDIM and with 2-carbon longer mycolic acids^15^, therefore implicating a more complex role for this chaperone in modulation of the cell surface for biofilm production in mycobacteria. In our work, transcription of *groEL1* was found to be significantly repressed during the transition from planktonic to intercellular aggregation (Supplementary Table 1), while it was significantly induced after substratum attachment (Supplementary Table 1). In agreement with our recent report^9^, genes involved in mycolic acid biosynthesis (*kasA*, *kasB*, *acpM*, *fas*) were significantly induced after substratum attachment (Supplementary Table 1), therefore confirming their upregulation during biofilm formation in BCG.

Taken together, the most significant changes that BCG experiences during the early stages of biofilm production were the downregulation of genes of the DosR-regulon during intercellular aggregation, and their upregulation upon substrate attachment. Further, we found a plausible explanation for the temporary requirement of genes already reported to be required for biofilm production (*PE1*, *nirB*, *lsr2*, *groEL1*, *kasA, kasB, fas,* and *acpM*) in mycobacteria.

We then evaluated the expression of *BCG0114*, *dosR*, *BCG0642c*, *ethR*, and *BCG3766c* by RT-qPCR. *dosR* and *BCG0114* were specifically downregulated at the intercellular aggregation step while both of them were upregulated at the substrate attachment stage (Supplementary Table 1), and as part of the DosR-regulon their role in regulating expression of other genes have been described^12,13,16^. *BCG0642c*, which encodes for a conserved hypothetical protein with a PhdYeFM antitoxin domain, was significantly upregulated only during intercellular aggregation and downregulated (*p* = 0.06) at the substrate attachment step (Supplementary Table 1). *ethR* was specifically downregulated during surface attachment, while *BCG3766c*, which encodes for a conserved hypothetical proline rich protein, was also was close to significant downregulation during surface attachment and almost reached the criteria to be considered as significantly affected during the intercellular aggregation step (FC 0.76, *p* = 0.022).

**Figure 1:**
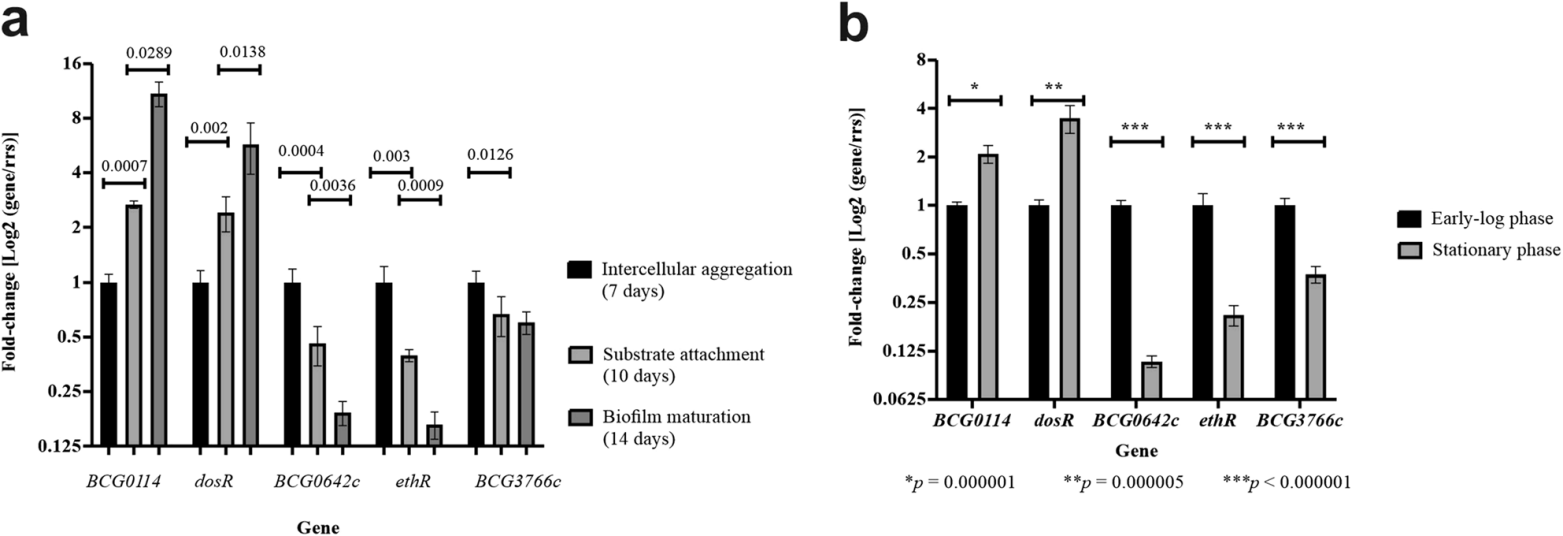
RT-qPCR analyses of a set of genes specifically regulated at the intercellular and substrate attachment steps during biofilm formation in BCG. Relative expression of genes at different steps during biofilm formation (**a**) and as planktonic cultures at early logarithmic and stationary phases (**b**). Error bars represent standard deviations of the mean from three biological replicates, each with technical duplicates, for six total data per gene. Brown–Forsythe and Welch ANOVA followed by Dunnett’s multiple comparison test was used to compare samples obtained from biofilms. Multiple *t* tests followed by Holm–Sidak comparison was used for samples obtained from planktonic cultures. Brackets encompass the comparisons for which statistically significant *p *values are shown on top of the bars depicting the means.

We sought to evaluate and validate the expression of these 5 selected genes by RT-qPCR at the same stages as we did for RNA-Seq analyses (Fig. 1a). Given that the mean Ct value for each gene of interest with respect to the reference gene, *rrs*, showed a statistically significant difference between the 24 h time point compared to the remaining ones (*p* < 0.0001 compared with 7 days, 10 days, and 14 days, one-way ANOVA followed by Tukey’s multiple comparison test), we were able only to compare differential gene expression as measured by RNA-Seq to that of the substrate attachment (10 days) versus intercellular aggregation (7 days) step as determined by RT-qPCR. Transcription of the reference gene, *rrs*, was found to be non-significantly different for biofilm samples (mean Ct values of 9.9, 9.7, and 10.7 at days 7, 10, and 14, respectively, with *p* values 0.9724 for the 10 vs. 7 days comparison, 0.2921 for the 14 vs. 7 days comparison, and 0.282 for the 10 vs. 14 days comparison, determined by the Brown–Forsythe and Welch ANOVA test followed by Dunnett’s post-hoc comparison). Similarly, *rrs* transcription was not statistically different for planktonic cultures, with mean Ct values of 8.9 (log phase) and 9.4 (stationary phase cultures, *p* = 0.1409 after a two-tailed, unpaired *t* test with Welch correction).

We found an agreement in expression for 4 out of 5 genes, with a slight variation in the magnitude of the change measured: *BCG0114* (FC = 6.72 by RNA-seq; FC = 2.66 by RT-qPCR), *dosR* (FC = 3.62 by RNA-Seq; FC = 2.4 by RT-qPCR), *ethR* (FC = 0.35 by RNA-Seq; FC = 0.39 by RT-qPCR), and *BCG3766c* (FC = 0.48 by RNA-Seq; FC = 0.67 by RT-qPCR). Only expression of *BCG0642c* showed discrepancy between RNA-Seq (2.1 mean FC) and RT-qPCR (0.46 mean FC).

Further evaluating gene expression by RT-qPCR we noticed that upregulation of *BCG0114* and *dosR*, or downregulation of *ethR* and *BCG0642c*, which both started at the substrate attachment step, were maintained during biofilm maturation (Fig. 1a). Whereas induction of *BCG3766c* was only found during substrate attachment with no further change during biofilm maturation (Fig. 1a).

To complete our gene expression analyses, we used RT-qPCR to monitor the expression of *BCG0114*, *dosR*, *BCG0642c*, *ethR*, and *BCG3766c* in planktonic cultures of BCG at early-log and stationary phase (Fig. 1b). Using early-log phase cultures as a reference, we observed that expression of all genes in stationary cultures followed the same pattern as they did during intercellular aggregation (*BCG0114* and *dosR* upregulated; *BCG0642c*, *ethR*, and *BCG3766c* downregulated). Hence, RT-qPCR validated differential expression for 4 out of 5 genes selected from RNA-Seq assays. Moreover, it showed that expression of the 5 selected targets in stationary phase planktonic cultures resembled the pattern found during intercellular aggregation.

### Phenotypic changes in multicellular BCG aggregates derived from increased expression of *dosR*, *BCG0114*, *BCG0642c*, *ethR*, and *BCG3766c*

Having confirmed that *dosR*, *BCG0114*, *BCG0642c*, *ethR*, and *BCG3766c* showed differential expression specifically at either the intercellular aggregation or substrate attachment steps, we next evaluated the effect of increasing their expression by inserting an additional single copy of each one of them into BCG Pasteur via pMV361, under the control of the strong promoter *hsp60*^17^. Expression of other genes from this promoter has already been shown by us to result in downstream changes at the transcriptomic^18^ and proteomic levels, altered infectivity in vitro^19^, and improved immunogenicity or vaccine efficacy in vivo^20^. Using this approach, we uncoupled gene transcription from the temporary differential expression observed in RNA-Seq and RT-qPCR assays.

**Figure 2:**
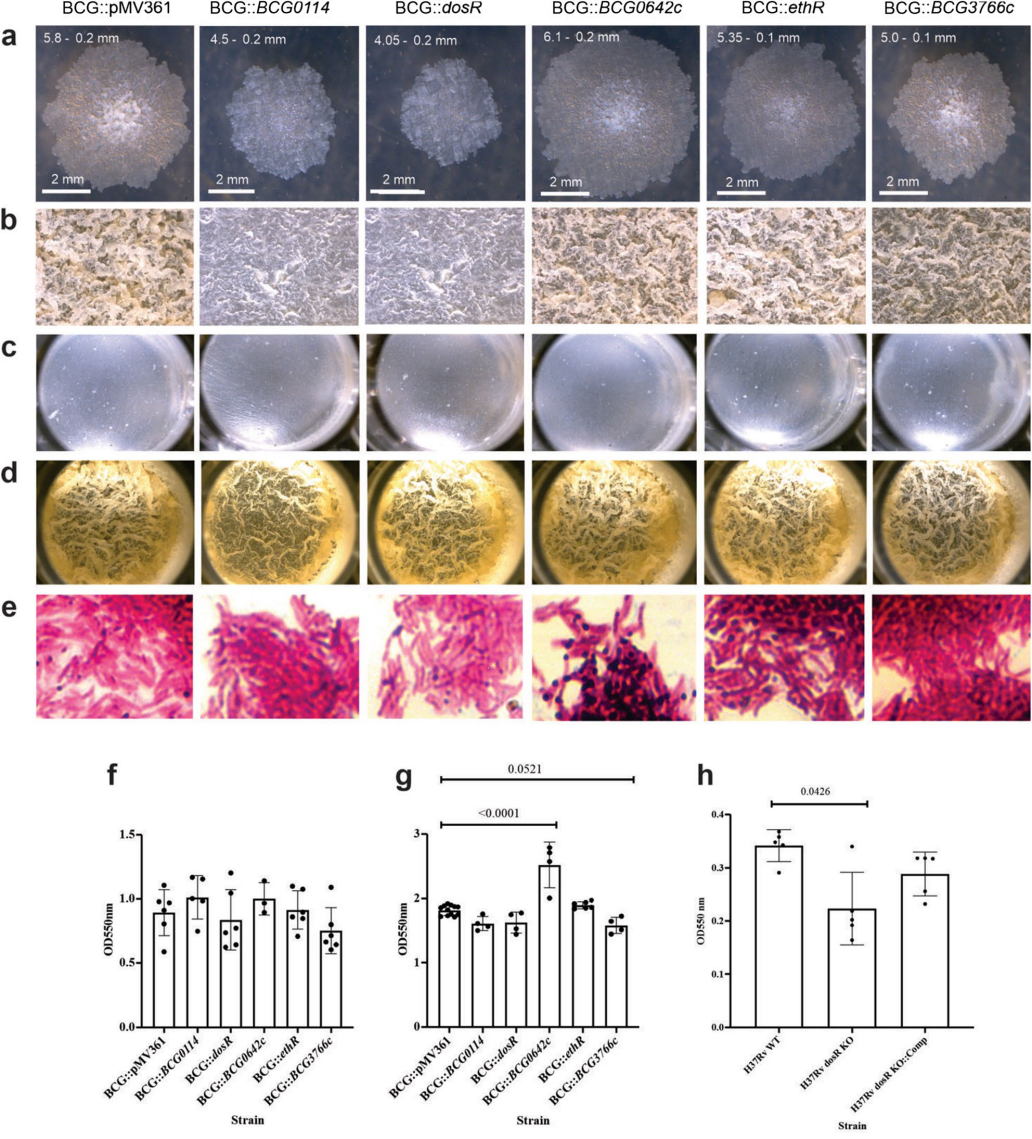
Phenotypic changes in multicellular BCG aggregates derived from increased expression of *dosR*, *BCG0114*, *BCG0642c*, *ethR*, and *BCG3766c*. BCG strains harboring each one of the indicated genes, under the control of the strong *hsp60* promoter, or the empty vector (pMV361), were evaluated for different multicellular phenotypes. Isolated, single colonies obtained after 3 weeks of incubation at 37 °C on 7H10 OADC agar plates (**a**). Surface pellicles formed in Sauton media with no detergent at 37 °C, 5% CO_2_, for 2-weeks in tissue culture flasks (**b**), 10 days (**c**) or 2 weeks in 48-well plates. Ziehl–Neelsen staining of the different BCG strains sampled from 2 weeks-old surface pellicles (**e**). Biofilm quantification of the different BCG at 10 days (**f**) or 2 weeks of culture in Sauton media, in 48-well plates. Biofilm quantification of *M. tuberculosis* H37Rv wild type, its isogenic deletion mutant devoid of the *Rv3134c-dosS-dosR* operon (H37Rv *dosR* KO) and its complemented derivative (H37Rv *dosR* KO::Comp) in 24-well plates using Sauton medium with no detergent. For colonies, a 2 mm scale bar is shown, with the mean diameter and standard deviation indicated (**a**). For surface pellicles, images at 10× are shown (**b**–**d**). For Ziehl–Neelsen staining, images at 100× are shown (**e**). All experiments were performed three different times, with duplicates (**a**), or four to six biological duplicates (**b**–**e**), and one representative image is shown in all instances. For biofilm quantification, the experiment was repeated independently three times and one representative set of results is shown; error bars represent standard deviations of the mean from four to six biological replicates, indicated as individual dots (**f**–**h**). One-Way ANOVA followed by Dunnett’s multiple comparison test was used to assess significance of changes between each recombinant BCG strain and wild type BCG harboring the empty vector (**f**–**g**), or among the three *M. tuberculosis* H37Rv strains (**h**). Brackets encompass the comparisons for which statistically significant *p* values are shown on top of the bars depicting the means.

Our initial assessment of the phenotypic changes in BCG was focused on those related to multicellular aggregates, such as colony morphology, surface pellicle appearance, Ziehl–Neelsen staining of biofilm samples, and biofilm formed at 10 (substrate attachment) and 14 days (biofilm maturation) as measured by crystal violet staining. Regarding colony morphology (Fig. 2a), we found morphological characteristics of the *M. tuberculosis* complex, such as: irregular form, waxy dry appearance, wrinkled and rough surface with irregular margins. We also found some differences among the strains, as follows: BCG strains harboring additional copies of *BCG0114* (BCG::*BCG0114*), and *dosR* (BCG::*dosR*) showed a grayish color, unlike the other strains that presented a light yellowish color. Also, both strains produced smaller and flatter colonies compared with the others. The strain with an additional copy of *BCG3766c* (BCG::*BCG3766c*) was the one most similar to the wild type strain harboring the empty vector (BCG::pMV361), the only difference was its smaller size, with both of them showing an irregular elevation in the center of the colony. Finally, strains harboring an additional copy of *ethR* (BCG::*ethR*) or *BCG0642c* (BCG::*BCG0642c*) were very similar to each other, the main difference being their size (Fig. 2a).

We also observed changes in mature surface pellicle appearance formed in tissue culture flasks, as both BCG::*BCG0114* and BCG::*dosR* produced smooth pellicles as opposed to the rugose ones produced by BCG::pMV361, BCG::*ethR,* BCG::*BCG0642c*, and BCG::*BCG3766c*, with slight variations among strains in apparent rugosity (Fig. 2b).

When we followed biofilm formation in multiwell plates, we noticed that at the substrate attachment step, no noticeable difference was found during biofilm formation (10 days, Fig. 2c), and minor variations in surface pellicles were observed when biofilms were mature (14 days, Fig. 2d), although a lower rugosity was consistently observed for both BCG::*BCG0114* and BCG::*dosR* cultures (Fig. 2d). No major changes were observed in acid-fastness or intercellular adherence of the different BCG strains in mature biofilms, with the exception of some metachromatic-like granules present in BCG::*ethR* and BCG::*BCG0642c* (Fig. 2e)*.*

Biofilm formed by any of the BCG strains at the substrate attachment step showed no quantitative difference compared with wild type BCG (Fig. 2f), and only BCG::*BCG0642c* produced more mature biofilm than wild type BCG (*p* < 0.0001) with BCG::*BCG3766c* almost reaching significance for a reduced production of this structure (*p* = 0.0521, One-Way ANOVA followed by Dunnett’s multiple comparison test) (Fig. 2g). We also evaluated the capacity to produce biofilms by *M. tuberculosis* strains with different *dosR* contents and found that a mutant lacking this operon (H37Rv *dosR* KO) was affected in its biofilm formation (*p* = 0.0426, One-Way ANOVA followed by Dunnett’s multiple comparison test) with capacity being restored to wild-type levels upon reinsertion of the gene into the chromosome (H37Rv *dosR* KO::Comp, Fig. 2h).

In summary, compared to wild type BCG, *hsp60*-driven expression of *dosR* and *BCG0114*, led to smaller colonies on agar, with changes in color and elevation, and also produced smoother surface pellicles with no quantitative effect on biofilm production. Moreover, deletion of *dosR* in *M. tuberculosis* H37Rv reduced biofilm production (Fig. 2). *hsp60*-driven expression of *BCG0642c* enhanced biofilm production with formation of metachromatic-like granules in acid-fast bacteria (Fig. 2). *hsp60*-driven expression of *BCG3766c* reduced colony size and surface pellicle rugosity, although less than *dosR* or *BCG0114*, and tended to decrease biofilm production (Fig. 2). *ethR* expression from *hsp60* did not produce any detectable difference in the assays performed here (Fig. 2).

### Phenotypic changes in planktonic BCG cells derived from increased expression of *dosR*, *BCG0114*, *BCG0642c*, *ethR*, and *BCG3766c*

**Figure 3:**
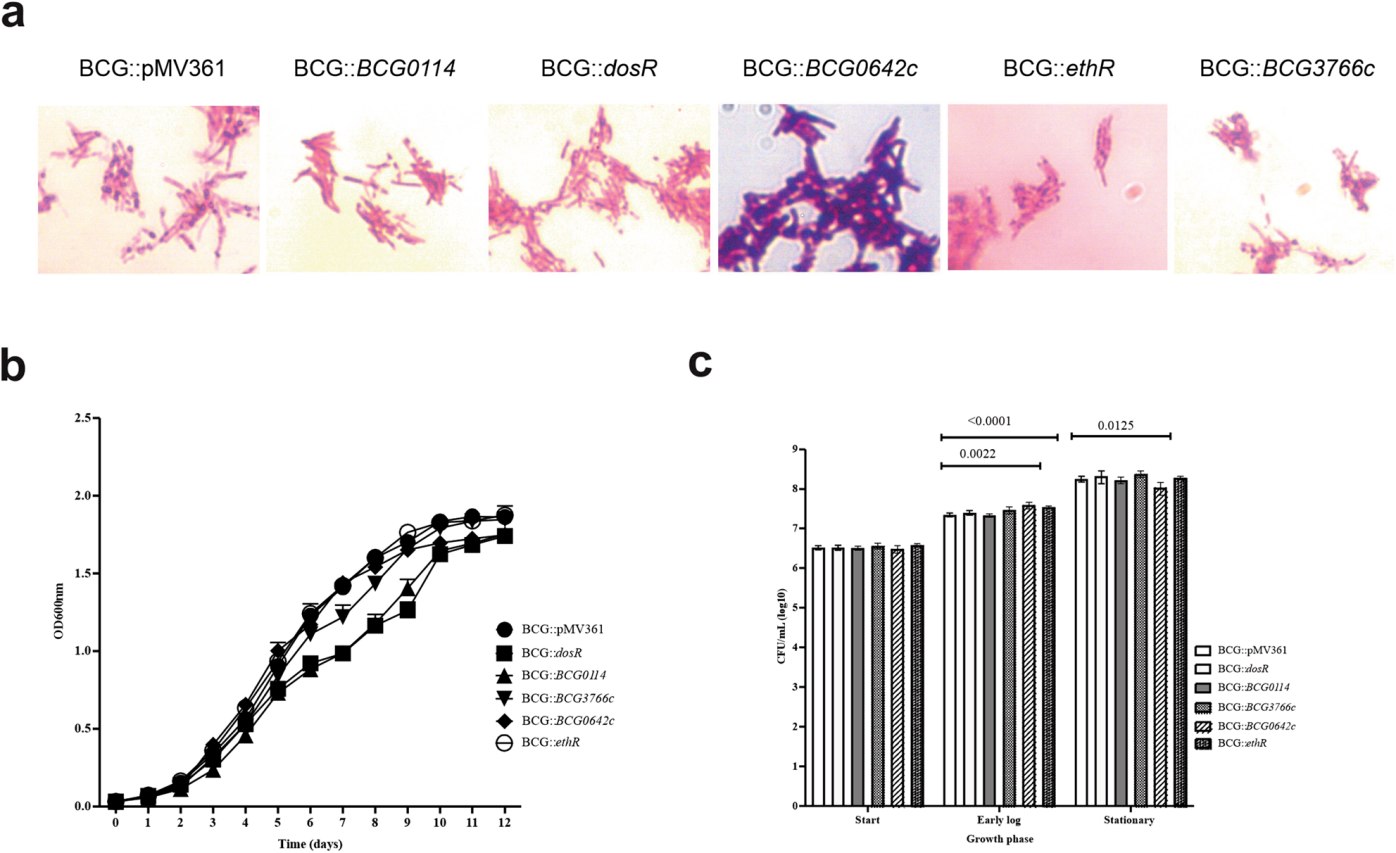
Phenotypic changes in planktonic BCG cells derived from increased expression of *dosR*, *BCG0114*, *BCG0642c*, *ethR*, and *BCG3766c*. Ziehl–Neelsen staining of the different BCG strains sampled from mid-log phase planktonic cultures in 7H9 OADC 0.05% Tween 80 (**a**). Growth (as OD600nm readings) of each recombinant BCG was compared with that of parental BCG harboring the empty vector pMV361 (BCG WT::pMV361) (**b**). Two-Way ANOVA followed by Dunnett’s multiple comparison test was used to compare apparent growth, using four biological duplicates; *p* values for the recombinant strains compared with BCG::pMV361 being described in results. Bacterial replication (CFU/mL) (**c**) was compared by 2-Way ANOVA followed by Tukey’s multiple comparison test, using four biological duplicates. Each experiment was repeated independently two times, and one representative result is shown. Brackets encompass the comparisons for which statistically significant *p* values are shown on top of the bars depicting the means.

After evaluating the effect of *hsp60*-driven expression of *dosR*, *BCG0114*, *BCG0642c*, *ethR*, and *BCG3766c* in BCG multicellular phenotypes, we next evaluated the effect of these genes in planktonic BCG cultures. We did not see any major difference in acid-fastness of early-log phase bacteria, except for the presence of metachromatic-like granules in BCG::*BCG0642c* (Fig. 3a) as it occurred for this strain in biofilm cultures (Fig. 2). An apparent tendency to form tight bundles was also noticed for BCG::*ethR* and BCG::*BCG3766c*, although this was not quantified.

Regarding the growth curve of planktonic BCG cultures, we monitored apparent growth by reading OD600nm every 24 h. There, we found that the most pronounced differences observed as an apparent growth delay as observed in OD600nm readings occurred when BCG had *hsp60*-driven expression of either *dosR* (significant differences in days 2 and 3, and from day 5 to day 12) or *BCG0114*, which significantly differed at all time points except at the start of the culture (Fig. 3b). Differences in OD600nm coincided with changes in doubling time for the BCG::dosR strain as compared with BCG::pMV361 at days 5 and 10 (46.94 ± 5.96 h vs. 35.6 ± 4.18, *p* = 0.0176; and 67.79 ± 11.42 h vs. 223.17 ± 22.96 h, *p* = 0.0086, respectively).

Even though OD600nm for BCG::BCG0114 suggested a marked growth defect as compared with wild type BCG harboring the empty vector (BCG::pMV361), doubling time indicated that there was indeed a significant difference between these strains, but only at day 6 of culture (BCG::pMV361, 55.17 ± 8 h vs. BCG::BCG0114, 88.65 ± 6.66 h; *p* = 0.0131). On the other hand, apparent growth of BCG::*ethR* significantly differed from wild type BCG in days 0 (start of the culture) and day 4 (early log-phase) (Fig. 3b), although their doubling time was different only at day 5 of culture (BCG::pMV361, 35.6 ± 4.18 h vs. BCG::ethR, 43.37 ± 5.04 h; *p* = 0.038).

OD600nm readings of *BCG0642c* significantly differed in days 0, 2, 4, and from day 10 to day 12 (stationary phase) (Fig. 3b); however, doubling time differed at day 1 (BCG::pMV361, 20.72 ± 1.33 h vs. BCG::BCG0642c, 29.05 ± 2 h; *p* = 0.0181) and day 3 only (BCG::pMV361, 21 ± 0.76 h vs. BCG::BCG0642c, 14.3 ± 1.97 h; *p* = 0.0067). *hsp60*-driven expression of *BCG3766c* resulted in significant differences in OD600nm readings at day 2, and from day 6 to day 8 (mid-log phase), with doubling time being significantly different at days 2 and 3 of culture (day 2, BCG::pMV361, 20.72 ± 1.33 h vs. BCG::BCG3766c, 26.08 ± 1.29 h; *p* = 0.0065. Day 3, BCG::pMV361, 21 ± 0.76 h vs. BCG::BCG3766c, 18.19 ± 0.75 h; *p* = 0.0111).

Next, we compared bacterial replication as colony-forming units (CFU) per milliliter at 3 stages: day 0 (start of the culture), day 4 (early-log phase), and day 10 (stationary phase). We found that despite the apparent growth delay produced in BCG upon *hsp60*-driven expression of *dosR* or *BCG0114*, specifically in the mid-log to stationary phase transition (Fig. 3b), no significant difference in terms of bacterial numbers was found in any of the three stages evaluated here (Fig. 3c). On the other hand, in agreement with differences found in the growth curve at day 4, BCG::*ethR* had higher CFU/mL than wild type BCG (*p* < 0.0001 for early-log cultures, Fig. 3c). Also in agreement with changes observed in growth curve was the replication of early-log and stationary phase cultures of BCG::*BCG0642c* (*p* = 0.0022 for early-log, and *p* = 0.0125 for stationary phases cultures, Fig. 3c). No significant change was detected in replication of BCG::BCG3766c compared with BCG::pMV361 (Fig. 3c).

## Discussion

Bacteria must accurately regulate growth and stress resilience. The formation of biofilms contributes to stress survival, since these dense multicellular aggregates, in which cells are embedded in an extracellular matrix of self-produced polymers, represent a self-constructed protective ‘niche’^21^ that yet remains metabolically active even after reaching maturity^22^.

In *M. tuberculosis* complex bacteria, biofilm production in vitro has been shown to harbor drug-tolerant bacteria^7^ and to be genetically linked to this phenotype^8^. Drug-tolerant mycobacteria may comprise a fraction of the population, as mature *M. tuberculosis* biofilms showed sensitivity to antibiotic treatment when assessed with microcalorimetry (IMC) and tunable diode laser absorption spectroscopy^22^. Drug tolerance has also been found to occur in ex vivo caseum^23^ and is thought to contribute to persistence after drug treatment in vivo^24^. In fact, the lungs of chronically infected mice harbored a subpopulation of nongrowing but metabolically active *M. tuberculosis*^25^.

Evidence reported over the last decade associate the capacity of *M. tuberculosis*-complex bacteria with virulence in ex vivo or in vivo models^26^. Pang et al.^14^ found several genes that were required for biofilm production using a “formation/no formation” readout, while Yang et al.^1^ utilized a genetic approach to propose a temporal order for development of mycobacterial biofilms using *M. smegmatis* as model.

In this work, we performed an unbiased, whole transcriptome analysis, aimed to find genes differentially expressed during intercellular aggregation and substrate attachment. This followed the rationale that upon affecting their expression levels, this may result in either major or subtle changes during biofilm formation by BCG. This contrasts with both Pang et al.^14^ strategy based on “formation/no formation” readout in microtiter plates, and the one used by Yang et al.^1^ that relied on a clever yet static approach, as these authors screened only one time point to look for *M. smegmatis* mutants with altered capacity to produce biofilms within a syringe-based model.

It is worth noting that we utilized a panel of 5 DE tools to identify gene expression changes. Selection of genes with potential relevance for the intercellular aggregation and substrate attachment steps during biofilm production by BCG were based on their up- or down-regulation by a twofold or greater change coupled with *p* < 0.05 after multivariate analysis. Using these criteria, we observed that the most significant changes that BCG experiences at the early stages of biofilm production are the downregulation of part of the DosR-regulon during intercellular aggregation, and their upregulation upon substrate attachment. Therefore, we selected 2 genes from this regulon: *dosR* itself, and *BCG0114* (homologous to *Rv0081*), and characterized the effects of the strong expression of these genes from the *hsp60* promoter both during multicellular and planktonic growth.

DosR has been shown to respond to oxygen limitation and to the presence of nitric oxide in vitro^12,13^. We found that only the absence of *dosR* in *M. tuberculosis*, as opposed to its increased expression in BCG, result in reduced biofilm production. We hypothesize that this may be explained by the fact of slow growing mycobacteria requiring DosR to metabolically adapt to low O_2_ levels as already reported^27^. This result is also in agreement with reduced *dosR* transcription and reduced biofilm production observed in a double mutant devoid of the exopolyphosphatases genes^28^ and also in a *mtrB* mutant^29^.

Changes in surface pellicle appearance during biofilm production upon *dosR* or *BCG0114* expression from *hsp60*, constitute a subtle phenotype that may go unnoticed when assessing transposon insertion mutant for “formation/no formation” readout, as we found no quantitative effect on biofilm production. The relevance of this biofilm-specific change for other in vitro or in vivo phenotypes remains to be determined. We contend that altering either *dosR* or *BCG0114* expression results in unique, biofilm-specific phenotypes, as during planktonic growth, their expression from *hsp60* delayed logarithmic growth (Fig. 3b) but with no effect on bacterial replication at the beginning, early-log, and stationary phases (Fig. 3c). This difference might be explained, at least to some extent, by differences in oxygen availability in biofilms as has been suggested^9^. In support of this hypothesis, it is worth noting that the already complex regulatory network utilized by *M. tuberculosis* to respond to hypoxia^16^ has just been refined using a comprehensive genome-wide transcription factor binding map and network topology analysis. This unraveled *M. tuberculosis* response during the adaptation to varying oxygen levels (normoxia, depletion, early-, mid-, and late hypoxia, and resuscitation) and contributed to further support the role of Rv0081 (BCG0114), DosR, and Lsr2 in adaptation to oxygen availability^30^, regulators that were differentially expressed at distinct stages of biofilm production in BCG (Table 1).

**Table 1:** Primers, plasmids and bacterial species used in this work

Sequence (5′–3′)	Primer name	Restriction site
**Primers used to amplify and clone ORFs under** ***hsp60***		
GGAGAATTCATGGAGTCCGAACCGCTG	BCG_0114-5F	EcoRI
CCCAAGCTTTTACGTGGCCGAGCC	BCG_0114-3R	HindIII
GTGCAGCTGTCATGGTAAAGGTCTTCTTGGTCG	devR-F-Pvu	PvuII
ACTAAGCTTCCTGTTGTCATGGTCCATCACCG	devR-RHd3	HindIII
ACTGAATTCATGTCTGCTACGATACC	BCG_0642c-5F	EcoRI
GCCAAGCTTTCACCACCG	BCG_0642c-3R	HindIII
CAAGAATTCATGCGACACATGAGT	BCG_3766c-5F	EcoRI
GGTAAGCTTTTACGGAGCGGG	BCG_3766c-3R	HindIII
GCTGAATTCATGACCACCTCC	EthR-5F	EcoRI
GAGAAGCTTTTAGCGGTTCTCG	EthR-3R	HindIII

Sequence (5′–3′)	Primer name	
**Primers used for real time qPCR**		
AGTCCGAACCGCTGTACAAG	BCG0114-5RT	
CAGCAGCTCCAAAATCCTGATC	BCG0114-3RT	
ATGGCAACGGCATTGAACT	dosR-F	
AGAATCGCATCTAGCATGGC	dosR-R	
GTAATCGCAGATCAGCAACG	rrs-F	
TTCGGGTGTTACCGACTTTC	rrs-R	
ATCTGAAACCCCAACACCAC	BCG_3766c-5RT	
ATAGCCGGTGAAGAAGATGACC	BCG_3766c-3RT	
TGGTCAATCAAGCCGACATG	ethR-5RT	
TCTCGAAGAACACGTTGATCCC	ethR-3RT	
ATCGCGCGAAAAACCATCTG	sigA-F	
TGGTGTAGTCGAACTTCTCCAC	sigA-R	
AGGAAATCGAGGTGCTCAAGG	BCG_0642c-5RT	
TCGCCCAGATTGGTGGTATC	BCG_0642c-3RT	

Plasmid	Description	
pMV361	Shuttle vector, kanamycin resistance marker, incorporates as single copy into *Mycobacterium* *attB* site, can drive gene transcription from the strong *hsp60* promoter	
pMV361-dosR	pMV361 with *dosR* inserted into PvuII/HindIII sites	
pMV361-BCG0114	pMV361 with *BCG0114* inserted into EcoRI/HindIII sites	
pMV361-BCG0642c	pMV361 with *BCG0642c* inserted into EcoRI/HindIII sites	
pMV361-BCG3766c	pMV361 with *BCG3766c* inserted into EcoRI/HindIII sites	
pMV361-ethR	pMV361 with *ethR* inserted into EcoRI/HindIII sites	

Strain	Genotype	
*M. bovis* BCG Pasteur	*Mycobacterium bovis* Karlson and Lessel (ATCC® 35734™) (hereafter referred as to BCG wild type -WT-)	
BCG WT::pMV361	BCG Pasteur with an insertion at *attB* of pMV361 (empty vector)	
BCG::dosR	BCG Pasteur with an insertion at *attB* of pMV361-dosR	
BCG::BCG0114	BCG Pasteur with an insertion at *attB* of pMV361-BCG0114	
BCG::BCG0642c	BCG Pasteur with an insertion at *attB* of pMV361-BCG0642c	
BCG::BCG3766c	BCG Pasteur with an insertion at *attB* of pMV361-BCG3766c	
BCG::ethR	BCG Pasteur with an insertion at *attB* of pMV361-ethR	
E. coli DH5α	F− φ80*lac*ZΔM15 Δ(*lac*ZYA-*arg*F)U169 *rec*A1 *end*A1 *hsd*R17(rK−, mK+) *pho*A *sup*E44 λ− *thi*-1 *gyr*A96 *rel*A1	

Underlined sequences indicate sites for endonuclease restriction

*BCG0642c*, which encodes for a conserved hypothetical protein with a PhdYeFM antitoxin domain, was significantly upregulated only during the intercellular aggregation step and tended towards downregulation (*p* = 0.06) at the substrate attachment step (Supplementary Table 1). To date, only the structure and function as an antitoxin of VapB4 (*Rv0596c*, the orthologous of *BCG0642c*) has been described^31^ but with no other role identified thus far. However, toxin-antitoxin modules have been shown to play a major role in persister formation in many model systems^32^, therefore it seems reasonable to find at least one of these genes as differentially expressed and contributing to biofilm production in BCG (Fig. 2). We acknowledge that a unique, biofilm-specific role for *BCG0642c* cannot be claimed at this point, given that its expression from *hsp60* also affected planktonic replication, positively during early-log phase, and negatively in stationary phase planktonic BCG cultures (Fig. 3).

*ethR* was specifically downregulated during surface attachment (Supplementary Table 1) but its expression from *hsp60* resulted in no change during biofilm production (Fig. 2) yet it favored growth and replication during early-log phase (Fig. 3). In this regard, a *M. bovis* BCG *ethA-ethR* KO mutant showed increased adherence to mammalian cells^33^ but its capacity to produce biofilm was not evaluated in that work. Nevertheless, another report stated that *ethR* did not participate in biofilm production in *M. tuberculosis*^34^, which seems in agreement with our findings in BCG.

The last gene we evaluated was *BCG3766c*, which encodes for a conserved hypothetical proline rich protein (Supplementary Table 1). This gene was significantly downregulated during surface attachment and was also downregulated during the intercellular aggregation step as well (FC 0.76, *p* = 0.022). This may explain why its expression from *hsp60* tended to reduce biofilm production (Fig. 2).

We also found differential expression for a number of other genes that we did not further evaluate in this work, including among the most significantly upregulated genes *sigE*, *fadE23* (fatty-acid-CoA ligase, involved in sulfolipid production)^10^, *hupB* (DNA binding protein), *BCG3929* (*Rv3866*, *espG*), *ppsC* (involved in PDIM synthesis), *BCG1191* (*Rv1130*, *prpD*, 2-methylcitrate dehydratase), and *BCG1826* (*Rv1794*, part of the ESX-5 secretion system)^11^. For *BCG3929* (*Rv3866*, *espG*), a deletion of *espG* in *M. marinum* reduced sliding motility and biofilm formation^35^. *BCG3929* upregulation during intercellular aggregation as compared to planktonic growth (Table 2) may explain the defect of the *M. marinum* mutant in biofilm formation.

**Table 2:** The thirty genes showing the highest differential expression (up- or down-regulated) at the intercellular aggregation step during biofilm production by BCG.

Gene_Name	Gene_ID	Log2fold	Avg_*p* value	Product
lppB	BCG_2567	2.7988	2.68E−08	Probable conserved lipoprotein *lppB*
BCG_3184c	BCG_3184c	2.5898	1.30E−15	Possible transcriptional regulatory protein (probably tetR-family), orthologous to *Rv3160c*. *Rv3160c* was involved in response to thioridazine (PLoS One. 2010 Apr 8;5(4):e10069. 10.1371/journal.pone.0010069)
PE22	BCG_2124	2.5607	5.75E−15	PE family protein
BCG_2486c	BCG_2486c	2.496	0.000174564	Conserved hypothetical protein. Contains a Protein Disulfide Oxidoreductase domain (7.33e−35)
BCG_3185c	BCG_3185c	2.4789	5.66E−19	Possible dioxygenase
BCG_1115	BCG_1115	2.4553	3.66E−16	Conserved hypothetical protein. Contains a DNA-binding beta-propeller fold protein YncE. (1.79e−34)
BCG_2748c	BCG_2748c	2.3981	3.53E−09	Conserved hypothetical protein. Contains a S-adenosylmethionine-dependent methyltransferases (SAM or AdoMet-MTase), class I domain (8.96e−66)
sigE	BCG_1281	2.2979	1.16E−16	Alternative RNA polymerase sigma factor *sigE*
BCG_0010c	BCG_0010c	2.2359	5.18E−21	Probable conserved membrane protein. It contains a 40 aa stretch (out of 111 aa) similar to bacterial plekstrin homology domain (2.65e−8)
BCG_0040c	BCG_0040c	2.2296	8.44E−20	Probable conserved membrane protein. It contains a 40 aa stretch (out of 111 aa) similar to bacterial plekstrin homology domain (2.65e−8)
lprR	BCG_2566	2.2247	1.51E−09	Probable conserved lipoprotein *lprR*
fadD23	BCG_3889	2.1317	3.07E−22	Probable fatty-acid-CoA ligase fadD23, involved in sulfolipid production (Microbiology. 2007 Sep;153(Pt 9):3133-40.)
PPE70	BCG_3183c	2.1152	2.70E−20	PPE family protein
hupB	BCG_3007c	2.1028	5.22E−07	Probable DNA-binding protein HU homolog *hupB*
BCG_0088	BCG_0088	2.1007	9.08E−15	Hypothetical protein
BCG_0221	BCG_0221	2.0983	1.45E−11	Conserved hypothetical protein
BCG_3929	BCG_3929	2.0163	8.42E−20	Conserved hypothetical protein. Orthologous Rv3866 (EspG). A deletion of *espG* in *M. marinum* reduced sliding motility and biofilm formation by *M. marinum* (Front Microbiol. 2018 May 30;9:1160. 10.3389/fmicb.2018.01160).
ppsC	BCG_2955	1.8996	9.31E−19	Phenolpthiocerol synthesis type-I polyketide synthase ppsC
rplK	BCG_0689	1.8485	4.98E−10	Probable 50S ribosomal protein l11 rplK
PPE53	BCG_3182c	1.848	9.55E−17	PPE family protein
recX	BCG_2749c	1.7905	5.94E−11	Regulatory protein *recX*
BCG_0653	BCG_0653	1.7809	3.40E−05	Hypothetical protein
BCG_1191	BCG_1191	1.7798	5.19E−05	Conserved hypothetical protein. Orthologous to *Rv1130*, PrpD, 2-methylcitrate dehydratase.
BCG_2192c	BCG_2192c	1.7692	7.07E−14	Possible transposase
lpqK	BCG_0436c	1.7462	6.92E−12	Possible conserved lipoprotein *lpqK*
BCG_1053	BCG_1053	1.7207	5.49E−09	Hypothetical protein
parA_1	BCG_3976c	1.7153	6.49E−14	Probable chromosome partitioning protein *parA*
BCG_0343	BCG_0343	1.7095	6.09E−11	Probable dehydrogenase/reductase
BCG_1826	BCG_1826	1.7054	1.42E−09	Conserved hypothetical protein. Orthologous to *Rv1794*, which is part of the ESX-5 secretion system (Mol Microbiol. 2012 Mar;83(6):1195-209. 10.1111/j.1365-2958.2012.08001.x.)
rpsK	BCG_3524c	1.704	1.04E−09	Probable 30S ribosomal protein S11 *rpsK*
tyrT	tyrT	-4.7515	0.00058183	tRNA-Tyr
BCG_0115	BCG_0115	-4.6431	2.78E−17	Probable oxidoreductase. Orthologous to *Rv0082*.
BCG_0112	BCG_0112	-4.1372	6.19E−31	Hypothetical protein. Orthologous to *Rv0079*, part of the DosR-regulon. Contains a 46 aa region (out of 273 aa) with Sigma 54 modulation/S30EA ribosomal protein C-terminus (2.2e−11)
BCG_1838c	BCG_1838c	-3.8293	2.20E−18	Hypothetical protein
BCG_3001	BCG_3001	-3.7853	9.73E−06	Possible conserved secreted protein
BCG_0114	BCG_0114	-3.5825	4.44E−09	Probable transcriptional regulatory protein. Orthologous to *Rv0081*. Part of the DosR-regulon.
hycD	BCG_0117	-3.3564	1.98E−11	Possible formate hydrogenlyase hycD (FHL)
BCG_0113	BCG_0113	-3.3198	1.60E−08	Conserved hypothetical protein. Orthologous to *Rv0080*. Part of the DosR-regulon. Contains a Pyridoxine 5'-phosphate (PNP) oxidase-like and flavin reductase-like proteins domain (1.66e−19)
gluU	gluU	-3.2724	0.000581961	tRNA-Glu
BCG_0410c	BCG_0410c	-3.1733	0.005478442	Conserved hypothetical protein. Xanthine dehydrogenase accessory factor. Xanthine and CO dehydrogenase maturation factor (3.83e−57)
EBG00001157338	EBG00001157338	-3.1712	0.000181053	tRNA
BCG_3454c	BCG_3454c	-3.165	1.60E−05	Conserved hypothetical protein. Type II toxin-antitoxin (TA) system Phd/YefM family antitoxin similar to *Mycobacterium tuberculosis* VapB antitoxins Orthologous to *Rv3385c*.
BCG_1725c	BCG_1725c	-3.1551	1.92E−11	Probable conserved ATP-binding protein ABC transporter
BCG_2178c	BCG_2178c	-3.1213	0.00184171	Conserved hypothetical protein. Contains Flavin-utilizing monoxygenases (4.46e−58)
BCG_2265	BCG_2265	-3.1205	3.06E−08	Conserved hypothetical protein
BCG_3157c	BCG_3157c	-3.1202	1.23E−14	Conserved hypothetical protein. Orthologous to *Rv3134c*, in operon with *dosR, part of the DosR-regulon*. Universal stress protein family.
serX	serX	-3.1175	0.000416156	tRNA-Ser
EBG00001157336	EBG00001157336	-3.081	0.000153611	tRNA
lysU	lysU	-3.0572	0.000223606	tRNA-Lys
serV	serV	-2.9977	0.000401639	tRNA-Ser
TB31.7	BCG_2650	-2.9848	6.22E−10	Conserved hypothetical protein TB31.7. Orthologous to *Rv2623*, part of DosR-regulon. Universal stress protein family.
EBG00001157351	EBG00001157351	-2.9789	5.40E−06	tRNA
iniB	BCG_0380	-2.9678	1.85E−17	Isoniazid inductible gene protein *iniB*
BCG_1777	BCG_1777	-2.9632	2.44E−17	Conserved hypothetical protein
argH	BCG_1698	-2.9341	8.39E−07	Probable Argininosuccinate lyase *argH*
BCG_2266	BCG_2266	-2.9043	7.57E−07	Conserved hypothetical protein. Contains a C-terminal 71 aa stretch similar to Superfamily of nucleases (8.51e−3)
hspX	BCG_2050c	-2.8879	9.61E−07	Heat shock protein hspX. Orthologous to R*v2031c*, alpha crystalin, part of the DosR-regulon.
BCG_2049c	BCG_2049c	-2.8578	1.28E−07	Conserved hypothetical protein. Orthologous to *Rv2030c*, part of the DosR-regulon. Contains N-terminal (aa 15-228) Phosphoribosyl transferase (PRT)-type I domain (4.14e−98) and another stretch (aa 223-663 out of 681) with Erythromycin esterase homolog domain (8e−155)
BCG_3154	BCG_3154	-2.8493	4.09E−09	Conserved hypothetical protein. Orthologous to *Rv3131*, part of the DosR-regulon.
BCG_3287c	BCG_3287c	-2.8072	3.32E−07	Conserved hypothetical protein
BCG_2624	BCG_2624	4.1276	0.009472594	Probable conserved integral membrane protein. 59 aa long.
BCG_0112	BCG_0112	4.0751	3.50E−24	Hypothetical protein. Orthologous to *Rv0079,* part of the DosR-regulon. Contains a 46 aa region (out of 273 aa) with Sigma 54 modulation/S30EA ribosomal protein C-terminus (2.2e−11)
BCG_2082	BCG_2082	3.5644	0.006797916	Conserved hypothetical protein
BCG_2051	BCG_2051	3.4061	4.19E−17	Conserved hypothetical protein Acg. Part of the DosR-regulon.
BCG_2653c	BCG_2653c	3.3755	2.16E−12	Conserved hypothetical protein. Orthologous to *Rv2626c*, part of the DosR-regulon. Contains CBS pair domain found in Hypoxic Response Protein 1 (HRP1) -like proteins (4.13e−50).
BCG_3157c	BCG_3157c	3.357	8.15E−16	Conserved hypothetical protein. Orthologous to *Rv3134c*, in operon with *dosR*, part of the DosR-regulon. Universal stress protein family.
BCG_1628	BCG_1628	3.324	9.27E−06	Conserved hypothetical protein
BCG_1777	BCG_1777	3.2859	6.02E−21	Conserved hypothetical protein. Orthologous to *Rv1738*, part of the DosR-regulon
BCG_1772c	BCG_1772c	3.1827	1.13E−12	Probable conserved transmembrane protein. Orthologous to *Rv1733c*, part of the DosR-regulon.
PE_PGRS42d	BCG_2505c	3.1391	0.000695518	PE-PGRS family protein [second part]
TB31.7	BCG_2650	3.1235	2.72E−10	Conserved hypothetical protein TB31.7. Orthologous to *Rv2623*, part of DosR-regulon. Universal stress protein family.
BCG_0115	BCG_0115	3.1231	1.05E−05	Probable oxidoreductase. Orthologous to *Rv0082*.
BCG_3454c	BCG_3454c	3.0372	0.000216715	Conserved hypothetical protein. Type II toxin-antitoxin (TA) system Phd/YefM family antitoxin similar to *Mycobacterium tuberculosis* VapB antitoxins
BCG_1838c	BCG_1838c	3.0323	1.20E−09	Hypothetical protein
BCG_2178c	BCG_2178c	3.032	0.008749493	Conserved hypothetical protein. Contains Flavin-utilizing monoxygenases (4.46e−58)
BCG_0614	BCG_0614	3.0014	4.54E−15	Conserved hypothetical protein. Orthologous to *Rv0569,* part of the DosR-regulon.
hspX	BCG_2050c	2.8196	1.61E−09	Heat shock protein hspX. Orthologous to R*v2031c*, alpha crystalin, part of the DosR-regulon.
thiE	BCG_0453c	2.8106	0.000357906	Probable thiamine-phosphate pyrophosphorylase *thiE*
BCG_0113	BCG_0113	2.7983	1.12E−06	Conserved hypothetical protein. Orthologous to *Rv0080*. Part of the DosR-regulon. Contains a Pyridoxine 5'-phosphate (PNP) oxidase-like and flavin reductase-like proteins domain (1.66e−19)
PE_PGRS13	BCG_0885	2.7947	3.02E−14	PE-PGRS family protein
BCG_0114	BCG_0114	2.7491	0.000116177	Probable transcriptional regulatory protein. Orthologous to *Rv0081*. Part of the DosR-regulon.
BCG_0168	BCG_0168	2.6959	3.97E−05	Pseudogene
BCG_2563	BCG_2563	2.6227	0.009558631	Hypothetical alanine rich protein
BCG_3001	BCG_3001	2.6148	0.009815904	Possible conserved secreted protein
BCG_3287c	BCG_3287c	2.6089	4.29E−05	Conserved hypothetical protein
BCG_0662	BCG_0662	2.592	1.93E−08	Probable integral membrane protein
BCG_3154	BCG_3154	2.5861	1.09E−07	Conserved hypothetical protein. Orthologous to *Rv3131*, part of the DosR-regulon.
BCG_2049c	BCG_2049c	2.5844	9.49E−08	Conserved hypothetical protein. Orthologous to *Rv2030c*, part of the DosR-regulon. Contains N-terminal (aa 15-228) Phosphoribosyl transferase (PRT)-type I domain (4.14e−98) and another stretch (aa 223-663 out of 681) with Erythromycin esterase homolog domain (8e−155)
leuT	leuT	2.5789	0.005156523	tRNA-Leu
BCG_3716c	BCG_3716c	2.5574	0.00164617	Probable conserved transmembrane protein. Contains Flp pilus assembly protein TadB domain (3.29e−41)
BCG_0010c	BCG_0010c	-4.5012	4.50E−31	Probable conserved membrane protein. It contains a 40 aa stretch (out of 111 aa) similar to bacterial plekstrin homology domain (2.65e−8)
BCG_0040c	BCG_0040c	-4.435	4.43E−17	Probable conserved membrane protein. It contains a 40 aa stretch (out of 111 aa) similar to bacterial plekstrin homology domain (2.65e−8)
BCG_3494c	BCG_3494c	-4.2797	3.95E−16	Hypothetical protein
lppU	BCG_2802c	-3.4282	1.19E−22	Probable lipoprotein *lppU*
BCG_2159c	BCG_2159c	-3.4146	1.35E−24	Hypothetical protein
BCG_2673c	BCG_2673c	-3.3701	2.34E−07	Hypothetical protein
BCG_3307c	BCG_3307c	-3.312	1.66E−11	Probable conserved transmembrane protein. It contains a 75 aa stretch (out of 111 aa) similar to bacterial plekstrin homology domain (4.36e−18)
BCG_0088	BCG_0088	-3.2898	6.03E−20	Hypothetical protein
rho	BCG_1357	-3.2069	1.45E−12	Probable transcription termination factor rho homolog
BCG_1104c	BCG_1104c	-3.1715	3.72E−28	Hypothetical protein
rpsM	BCG_3525c	-3.1288	2.36E−19	Probable 30S ribosomal protein S13 *rpsM*
BCG_3139	BCG_3139	-3.09	7.74E−06	Conserved hypothetical protein. tRNA(Arg) A34 adenosine deaminase TadA domain (1.47e−34)
BCG_1926c	BCG_1926c	-3.0538	0.000420994	Conserved hypothetical protein. S-adenosylmethionine-dependent methyltransferases domain (6.49e−22)
BCG_1350	BCG_1350	-3.0417	2.98E−05	Hypothetical protein
BCG_1001c	BCG_1001c	-3.039	0.000254116	Probable mycolyl transferase. 76 aa, similar to FbpC.
BCG_2748c	BCG_2748c	-3.0182	6.70E−24	Conserved hypothetical protein. Contains a three-Cys-motif partner protein domain (8.96e−66)
BCG_1115	BCG_1115	-2.9826	5.71E−10	Conserved hypothetical protein. Contains a DNA-binding beta-propeller fold protein YncE (1.79e−34)
dnaA_2	BCG_0031	-2.9643	3.00E−24	Chromosomal replication initiator protein *dnaA*
PPE70	BCG_3183c	-2.9502	6.02E−20	PPE family protein
BCG_3911	BCG_3911	-2.9473	1.83E−08	Probable conserved transmembrane protein. Putative Ca2+/H+ antiporter (2.81e−61)
BCG_3929	BCG_3929	-2.9222	4.01E−22	Conserved hypothetical protein. Orthologous *Rv3866* (EspG). A deletion of *espG* in *M. marinum* reduced sliding motility and biofilm formation by *M. marinum* (Front Microbiol. 2018 May 30;9:1160. 10.3389/fmicb.2018.01160).
BCG_2192c	BCG_2192c	-2.9092	9.86E−14	Possible transposase
lpqK	BCG_0436c	-2.9037	3.46E−08	Possible conserved lipoprotein *lpqK*
rpsK	BCG_3524c	-2.8992	1.14E−17	Probable 30S ribosomal protein S11 *rpsK*
dnaA_1	BCG_0001	-2.8632	5.63E−22	chromosomal replication initiation protein *dnaA*
BCG_1987c	BCG_1987c	-2.8213	1.10E−10	Hypothetical protein
BCG_0078c	BCG_0078c	-2.8174	1.00E−14	Conserved hypothetical protein. N-terminal (74 aa) with Transcriptional regulator PadR-like family domain (3.67e−22)
rpoA	BCG_3522c	-2.7989	2.70E−08	Probable DNA-directed RNA polymerase (alpha chain) *rpoA*
BCG_3899	BCG_3899	-2.7944	8.14E−08	Conserved hypothetical protein. Contains Minimal MMP-like domain (6.69e−50)
lprR	BCG_2566	-2.7685	1.32E−05	Probable conserved lipoprotein *lprR*. Orhtologous to *lppA*

Biofilm formation by BCG in the presence of the histone methyltransferase SUV39H1 was reduced, an effect proposed to occur via trimethylation of HupB^36^. This suggests a positive effect for this DNA-binding protein for biofilm production, and it is in agreement with *hupB* upregulation during intercellular aggregation (Table 2).

*Rv3385c* (orthologous to *BCG3454c*) was shown to be repressed in mature biofilms formed upon exposure to DTT as compared to late-log cultures of *M. tuberculosis*^37^. Redox conditions intervene in modulating *M. tuberculosis* pathogenesis, including activity of DosR^38^, which, to add further complexity to the mechanisms of gene regulation driven by this transcriptional regulator, was recently shown to be positively affected by c-di-GMP binding in *M. smegmatis* in response, precisely, to oxidative stress^39^.

Biofilm-specific proteins were recognized by antibodies present in sera from *M. tuberculosis* infected guinea pigs^40^. Of the antigenic proteins reported in that study, we found *ceoB* and *BCG2013* (*Rv1996*) significantly repressed during the transition from planktonic to intercellular aggregation (Supplementary Table 3), while they were significantly induced after substratum attachment (Supplementary Table 4). Moreover, *BCG2232* (*Rv2216*) and *TB39.8* (*BCG0050c*, *Rv0020c*, *FhaA*) were affected specifically after substratum attachment (FC = 1, and − 0.74 Log_2_, respectively, Supplementary Tables 4 and 5). The fact that some biofilm-specific proteins that were recognized in vivo had their encoding genes differentially expressed during biofilm production in vitro by BCG further strengthen the notion of biofilms mimicking aspects found during TB pathogenesis^26^. Taken together, our results show that *dosR* and *BCG0114* were expressed in a temporal order during mycobacterial biofilm formation to produce biofilm-specific changes, which most likely are triggered in response to varying oxygen levels within biofilms. Furthermore, we also provide a potential explanation for a stage dependent expression of additional genes previously reported to contribute to biofilm production in mycobacteria and suggest new targets that can be assessed for their particular contribution to this phenotype.

## Methods

### Bacterial strains, growth conditions and RNA extraction

*M. bovis* BCG Pasteur strain (ATCC 35734) or *M. tuberculosis* H37Rv and derivatives with deletion of the *Rv3134c-dosR-dosS* operon (referred to as H37Rv *dosR* KO in Fig. 2) and its complemented strain (referred to as H37Rv *dosR*::KO::Comp in Fig. 2)^27^ were used in this study. Planktonic cultures were performed in Middlebrook 7H9 liquid media (BD) with 10% OADC, 0.2% glycerol, 25 µg/mL of kanamycin, at 37 °C, 100 rpm. Serial dilutions of samples were followed by plating onto Middlebrook 7H10 agar plates supplemented with 10% OADC, 0.5% glycerol, and 25 µg/mL kanamycin served to determine colony-forming units per milliliter (CFU/mL). Biofilms (which include bacteria attached to the plastic wells and surface pellicles) for RNA extraction of BCG strains were cultured in Sauton media as already reported^9^. After 1, 7, 10 and 14 days of incubation, two culture flasks were used to harvest, with a scraper, the whole surface pellicle and biofilm attached to the wells (these samples are referred to as “biofilms”), and transferred into 50 mL tubes that were immediately frozen at − 70 °C. From frozen samples, we proceeded to perform RNA extraction and purification as already reported^41^, to ship these samples to Arizona State University for RNA-Seq analyses. The experiment was repeated three (7, 10, and 14 days cultures) or four times (24 h cultures, because of the low biomass present at this time point), to produce independent replicates.

### Temporal expression profiling during biofilm production by BCG

RNA was used to prepare cDNA using Nugen’s Ovation RNA-Seq System via single primer isothermal amplification (Catalogue # 7102-A01) and automated on the (BRAVO NGS liquid handler from Agilent). cDNA was quantified on the Nanodrop (Thermo Fisher Scientific). Using Kapa Biosystem’s DNA Hyper Plus library preparation kit, (KK8514) cDNA was enzymatically sheared to approximately 150 bp fragments, end repaired and A-tailed. Adapters with unique indexes compatible with Illumina (IDT #00989130v2) were ligated on each sample individually, then, these were cleaned using Kapa pure beads (Kapa Biosciences, KK8002), followed by amplification with Kapa’s HIFI enzyme (KK2502). Using Agilent’s Tapestation, we analyzed fragment size of each library, and quantified them by qPCR (KAPA Library Quantification Kit, KK4835) on a Quantstudio 5 (Thermo Fisher Scientific). Next, we multiplex pooled and sequenced a 2 × 75 flow cell on the NextSeq500 platform (Illumina) at the ASU’s Genomics Core facility.

### RNA-seq analysis

Raw FASTQ read data were processed using the R package DuffyNGS as described previously^42^. Briefly, raw reads were passed through a 3-stage alignment pipeline: (1) a prealignment step, to remove unwanted transcripts, such as rRNA; (2) a main genomic alignment step against the genome of interest; and (3) a splice junction alignment step, compared with an index of standard and alternative exon splice junctions. Reads were aligned to *M. bovis* BCG str. Pasteur (1173P2) with Bowtie2^43^, using the command line option “very-sensitive.” BAM files from stages (2) and (3) were combined into read depth wiggle tracks that recorded both multiply mapped and uniquely mapped reads to each of the forward and reverse strands of the genome of reference at single-nucleotide resolution. Next, gene transcript abundance was measured by summing total reads found inside annotated gene boundaries, expressed as both RPKM and raw read counts. RNA-seq data (raw fastq files and read counts) have been deposited in the GEO repository under accession number GSE150030.

### Differentially expressed genes

A panel of 5 DE tools was used to identify gene expression changes between 1-week old biofilm samples and 24-h samples (to determine genes affected or necessary for intercellular aggregation or cell-to-cell attachment) or 10-day old biofilm samples and 1-week old biofilm samples (to determine genes affected or necessary for substratum attachment to start building up the mature biofilm). The tools included (1) RoundRobin (in-house); (2) RankProduct^44^; (3) significance analysis of microarrays (SAM)^45^ ; (4) EdgeR^46^; and (5) DESeq2^47^. Appropriate default parameters were used to call each DE tool and operated on the same set of transcription results. For RankProduct, RoundRobin, and SAM, we used RPKM abundance units and for DESeq2 and EdgeR, raw read count abundance units were used. Next, we combined gene DE rank positions across all 5 DE tools. This consists of averaging a gene’s rank position in all 5 results, using a generalized mean to the 1/2 power, to yield the gene’s final net rank position. Similarly, explicit measurements of differential expression (fold change -FC-) and significance (*p *value) determined by each DE tool were combined via appropriate averaging (arithmetic and geometric mean, respectively). Genes with averaged absolute Log_2_ FC bigger than one and multiple hypothesis adjusted *p *value below 0.05 were considered differentially expressed. When gene function was predicted for those Conserved Hypothetical Proteins (CHP) and Hypothetical Proteins (HP) found among the 30 most up- or down-regulated genes during biofilm formation, we took the whole predicted protein sequence from BCG Pasteur 1173P2 (https://www.genome.jp/kegg-bin/show_organism?org=mbb), and used to search for Conserved Domains using the CD-Search tool from NCBI (https://www.ncbi.nlm.nih.gov/Structure/cdd/wrpsb.cgi).

### Reverse transcription coupled to quantitative PCR (RT-qPCR)

In order to independently determine the relative expression of genes selected from RNA-Seq experiments, total RNA samples obtained from additional cultures grown as biofilms (days 1, 7, 10, and 14) or planktonic cells (OD600nm 0.3 and ≈ 1.7) were quantified by UV spectroscopy and shipped to Centro Médico Siglo XXI for real time qPCR assays.This was conducted essentially as described^9^. Briefly, cDNA was synthesized using 500 ng of RNA, 0.2 µg/µl of random hexamer primers and 2 U/µl of Revertaid M-MulV-RT (Thermo Scientific). Specific gene primers are listed in Table 1. Control reactions were run in all experiments, with no transcript detected. Quantitative real-time PCR was performed in a LightCycler 480 instrument (Roche) and 16S rRNA (*rrs*) was used as a reference gene for normalization. The relative gene expression was calculated using the 2^−ΔΔCt^ method^48^.

### Construction of recombinant plasmids to drive expression of selected genes under the strong promoter *hsp60*

Primers used to amplify the open reading frame (ORF) of each one of the selected genes, recombinant plasmids generated, bacterial strains used in this work, and primers used for real time qPCR are indicated in Table 1. ORFs were amplified from genomic DNA obtained from BCG Pasteur by PCR using high fidelity Q5 DNA polymerase (New England Biolabs), digested with specific endonucleases and cloned under the *hsp60* promoter in pMV361^17^. Identity and fidelity of the ORFs was confirmed by DNA sequencing. Amplify4 for MacOS was used to design and test primers. DNA Strider 3.0 for MacOS was used for virtual cloning and plasmid characterizations. Sequence fidelity of cloned ORFs was evaluated using BLAST alignments both locally with DNA Strider 3.0 for MacOS and by direct comparison with genome sequences of BCG Pasteur 1173P2 (https://www.genome.jp/kegg-bin/show_organism?org=mbb). Recombinant plasmids were transformed into BCG by electroporation and selected on Middlebrook 7H10 (BD) OADC (BD-BBL) with 0.5% glycerol (Sigma) agar plates containing 25 µg/mL of kanamycin (Sigma).

### Growth curve and bacterial enumeration

Planktonic cultures were performed in Middlebrook 7H9 liquid media (BD) with 10% OADC, 0.2% glycerol, 25 µg/mL of kanamycin, at 37 ºC, 100 rpm, with a starting OD600nm of 0.03, to read OD600nm every 24 h. When each strain reached OD600nm of ≈ 0.3 (early-log), ≈ 0.6 (mid-log) and ≈ 1.7 (stationary phase), we took 1 mL samples. Serial dilutions of these samples were followed by plating 50 µL aliquots onto Middlebrook 7H10 agar plates supplemented with 10% OADC, 0.5% glycerol, and 25 µg/mL kanamycin served to determine colony-forming units per milliliter (CFU/mL).

### Ziehl–Neelsen staining

We used 10 µL aliquots from mid-log phase samples (planktonic cultures) or a loop of fully mature biofilms (2 weeks-old) taken with a disposable inoculating loop (BD 220,215). After fixation, staining was performed with a Ziehl–Neelsen kit (Hycel, https://www.hycel.com.mx/productos/kit-colorantes/) according to the manufacturer´s instructions.

### Quantification of biofilm by crystal violet staining

All mycobacterial strains were cultured in Sauton media, started at OD600nm 0.03, in 24-well (*M. tuberculosis*) or 48-well non-treated tissue culture plates (BCG strains), and were incubated at 37 °C, 5% CO_2_. Each strain was inoculated into 6 different wells, and experiments were repeated three times for statistical analysis. After 10 days (BCG strains) and 14 days (BCG strains and *M. tuberculosis* strains) of incubation, liquid media was removed and the whole surface pellicle and biofilm attached to the wells (these samples are referred to as “biofilms”) was maintained. Plates were baked at 30 °C for 24 h and 1 ml of 100% methanol was added to each well and incubated at room temperature for 15 min. Then, methanol was removed, and plates were dried at 37 °C for 15 min. Crystal violet (CV) was added to each well and incubated at 37 °C for 5 min. CV was removed and each well was washed four times with deionized water. Plates were dried at 37 °C for 15 min. Dye was extracted with 30% acetic acid for 15 min at 37 °C. Then the extract from each well was diluted 1:10 (*M. tuberculosis*), 1:20 (BCG, 10 days cultures), or 1:40 (BCG, 14 days cultures) in 30% acetic acid and read for OD550nm.

#### Statistics

Data distribution for qPCR, CFU, and biofilm quantification were analyzed using the Anderson–Darling and Shapiro–Wilk tests, and found to follow a normal distribution in all instances. Growth (as OD600nm readings) of rBCG strains was compared with that of parental BCG harboring the empty vector pMV361 (BCG WT::pMV361) using Two-Way ANOVA followed by Dunnett’s multiple comparison test. Growth (as doubling time) was compared by One-Way ANOVA (logarithmic phase cultures) or Brown–Forsythe and Welch ANOVA (stationary phase cultures) followed by Dunnett’s multiple comparison tests. Bacterial replication (CFU/mL) was compared by 2-Way ANOVA followed by Tukey’s multiple comparison test. For quantification of biofilms, statistical significance was determined using One Way ANOVA followed by Dunnett’s test for multiple comparison. For RT-qPCR analyses, Brown-Forsythe and Welch ANOVA followed by Dunnett’s multiple comparison test was used for comparing biofilm samples; multiple *t* tests followed by Holm–Sidak multiple comparison test was used for comparing planktonic samples. GraphPad Prism 8 for MacOS was used for performing statistical analyses. Assays were conducted in three independent times, and the number of replicates per experiment is indicated in each figure legend.

## Supplementary information

Supplementary Table 1.

Supplementary Table 2.

Supplementary Table 3.

Supplementary Table 4.

Supplementary Table 5.
